# The Role of Antigen Processing and Presentation in Cancer and the Efficacy of Immune Checkpoint Inhibitor Immunotherapy

**DOI:** 10.3390/cancers13010134

**Published:** 2021-01-04

**Authors:** Anastasia Mpakali, Efstratios Stratikos

**Affiliations:** 1 National Centre for Scientific Research Demokritos, Agia Paraskevi, 15341 Athens, Greece; 2 Laboratory of Biochemistry, Department of Chemistry, National and Kapodistrian University of Athens, Panepistimiopolis Zographou, 15784 Athens, Greece

**Keywords:** cancer, immunotherapy, adaptive immunity, antigen presentation, antigen processing, immune checkpoint inhibitor(s), major histocompatibility complex, human leukocyte antigens, antigenic peptide, neoantigen, aminopeptidase

## Abstract

**Simple Summary:**

A new class of drugs, termed Immune Checkpoint Inhibitors, has revolutionized cancer therapy during the last few years. Unfortunately, these drugs are only effective for a subset of patients and cancer types. Recent work has suggested that how well cancer cells present some of their molecules to the immune system is critical for patient responses to immunotherapy with immune checkpoint inhibitors. Here, we review the role of the biochemical pathway of antigen presentation in cancer and discuss how it can be modulated to enhance the efficacy of cancer immunotherapy.

**Abstract:**

Recent clinical successes of cancer immunotherapy using immune checkpoint inhibitors (ICIs) are rapidly changing the landscape of cancer treatment. Regardless of initial impressive clinical results though, the therapeutic benefit of ICIs appears to be limited to a subset of patients and tumor types. Recent analyses have revealed that the potency of ICI therapies depends on the efficient presentation of tumor-specific antigens by cancer cells and professional antigen presenting cells. Here, we review current knowledge on the role of antigen presentation in cancer. We focus on intracellular antigen processing and presentation by Major Histocompatibility class I (MHCI) molecules and how it can affect cancer immune evasion. Finally, we discuss the pharmacological tractability of manipulating intracellular antigen processing as a complementary approach to enhance tumor immunogenicity and the effectiveness of ICI immunotherapy.

## 1. The Immune System and Cancer

The interplay between the immune system and cancer termed “cancer immunoediting” is a dynamic and continuously evolving process in which immune responses can eradicate tumor cells but also promote tumor progression through selective pressures [1,2]. The temporal evolution of the immune system–cancer interaction is usually considered to consist of at least three phases termed elimination, equilibrium, and escape [3]. During the elimination phase, often at the initial stages of carcinogenesis, the immune system aggressively destroys newly formed cancer cells. If this attack is successful in eliminating all pre-cancerous and cancerous cells, no clinically detectable tumors are formed. Failure to eliminate all cancer cells however can result in establishment of an equilibrium phase in which the immune system controls tumor growth but cannot fully eliminate it [4]. This phase is considered to include some degree of immune evasion and can lead to a strong selective pressure on cancer cells to mutate in ways to further avoid the immune surveillance either by becoming less immunogenic or by inducing a localized immunosuppressive state. Success in these processes leads to the escape phase that allows out-of-control cancer cell growth and the appearance of clinically visible tumors that are characterized by different mechanisms and magnitudes of immune evasion and suppression [5].

## 2. Mechanisms of Cancer Immune Evasion and the Role of Immune Checkpoints

Tumors can attempt to evade cellular immune responses either by excluding T cells from the tumor microenvironment (TME) or by establishing equilibrium with T cells that successfully migrate to the tumor [6]. The former mechanism, termed innate evasion, includes accumulation of defects in T cell priming and reduced intratumoral trafficking through aberrant cell-intrinsic signaling events. Such events include activation of the Wnt/β-catenin pathway [7], loss of function of PTEN [8], c-Myc signaling dependent activation [9], and loss of LKB1 signaling [10]. The latter mechanism, termed adaptive immune evasion can emerge from the selection of tumor cell clones that have progressively reduced their immunogenicity through the loss of expression of key tumor-specific antigens and/or the accumulation of mutations in genes involved in immune recognition [11,12,13]. Notably, loss of MHCI expression is a common mechanism utilized by tumors attempting to evade T cell cytotoxic responses [14,15]. Loss of immune signaling can also synergize with loss of antigenicity by interfering with interferon generation and function [16,17]. In general, synergism between innate and adaptive immune evasion can result to a major therapeutic challenge that may only be overcome by combining separate approaches that, in tandem, address problems in both the TME as well as tumor immunogenicity.

T-cell-mediated immunity is regulated by a balance between stimulatory and inhibitory signals [18]. After encountering their cognate antigen, T cells, via their CD28 receptor, are activated by stimulatory signals in the context of antigen presenting cells (APCs) in order to attack and eliminate cancerous cells. However, their inflammatory activity must then be diminished to preserve immune homeostasis. The inhibitory signals are provided by molecules called immune checkpoints (ICs) that, when activated, suppress T cell activity [19]. These molecules are receptors located on the surface of T lymphocytes that regulate the extent and duration of physiological immune responses and therefore limit tissue damage and maintain self-tolerance. Several inhibitory checkpoint molecules have been discovered to date, such as CTLA-4 (cytotoxic T lymphocyte-associated protein 4)**,** PD-1 (programmed cell death protein 1), LAG-3 (lymphocyte activation Gene-3), TIM-3 (T-cell immunoglobulin and mucin-domain containing 3), TIGIT (T cell immunoglobulin and ITIM domain), VISTA (V-Domain Ig Suppressor of T-Cell Activation), B7-H3, BTLA (B and T lymphocyte attenuator 4), and Siglec-15 [20]. CTLA-4 and PD-1 are the most well studied and play central roles in state-of-the-art immunotherapy strategies. CTLA-4 is upregulated immediately after TCR engagement and through its competition with the co-stimulatory molecule CD28 for the B7 ligands (CD80/B7.1 and CD86/B7.2) of the APCs, it limits autoreactive T cells early at their activation stage leading to immune tolerance and prevention of autoimmunity [21,22]. Besides its surface expression being upregulated, additional CTLA-4 is recruited to the immunologic synapse via intracellular vesicles to further dampen T cell receptor (TCR) signaling [23]. Through the recruitment of phosphatases, CTLA-4 interferes with the TCR-induced stop signal for stable immune conjugate formation, leading to fewer contact periods between T cells and APCs and finally to decreased T cell priming and proliferation. The CTLA-4 suppressive functions can be also mediated by regulatory T cells (T_regs_), as it is expressed on their surface [24,25]. Recently, it was shown that CTLA-4 can deplete, through trans-endocytosis, available CD80 and CD86 ligands from the membranes of neighboring APCs to prevent their interaction with CD28 on T cells [26]. PD-1 is also expressed on activated T cells but acts at later stages of an immune response and interferes with previously activated T cells. If the stimulating antigen is cleared, PD-1 expression levels decrease on responding T cells whilst in the opposite case, its expression remains elevated. PD-1 has two tyrosine motifs in its cytoplasmic tail. Through its interaction with its ligands, PD-L1 and PD-L2, PD-1 is phosphorylated at these tyrosine residues, which leads to phosphatase recruitment. These phosphatases can then dephosphorylate downstream kinases and antagonize positive signals that take place through TCR and CD28, affecting TCR-mediated downstream signaling. The final outcome is impaired T cell activation, survival, cytokine production, and altered metabolism [27]. Sustained expression of PD-1 is considered to render T cells exhausted. PD-L1 expression is induced in response to inflammatory cytokines, such as IFNγ, and thus PD-1 regulation of T cell activity occurs in response to cytolytic and effector T cell function [28].

Signaling through PD-1 is a common mechanism that tumors utilize in order to put T cells in check and escape immunosurveillance. This can be achieved by upregulating PD-L1 expression on tumor cells themselves or on stromal and immune cells in the TME [25,29]. In mouse tumor cells, the upregulated expression of PD-L1 has been associated with impaired T cell mediated antitumor responses [30,31,32]. The combination of these findings with the recognition of ICs as negative regulators of T cell activation, gave rise to the idea that blocking the inhibitory action of ICs on T cells by using specific monoclonal antibodies, could improve T cell functions and enhance immune responses against cancer [33]. These pioneering cancer therapy approaches have now shifted the focus from attacking the tumor to assisting the host’s immune system to attack cancer cells. The presence of pre-existing cancer-specific T cells capable of recognizing tumor-specific antigens and neoantigens has been considered a necessary premise for this therapeutic approach [34]. However, although for many years the main mechanism of action of ICIs has been considered to be the re-activation of primed T cells, recently the role of novel T cells that are primed and recruited to the tumors after the initiation of immunotherapy has been emerging [35,36]. Regardless of the exact mechanism, the main advantage of ICI therapy is that it can induce durable responses representative of tumor-specific immunological memory formation [37]. Several antibody ICIs have already been FDA-approved since 2011 and have shown clinical efficacy in many cancer types (Table 1) [38].

CTLA-4 blockade aims to induce robust activation of tumor reactive T cells. By sterically hindering the interaction of CTLA-4 receptor with B7 ligands, it leads to unrestrained CD28-mediated positive co-stimulation of T lymphocytes. The co-crystal structure of the first approved antibody against CTLA-4, ipilimumab, in complex with CTLA-4 revealed that the epitope recognized by ipilimumab overlaps with the B7 interaction domain [39]. Additionally, CTLA-4 blocking promotes antitumor responses through the deletion of T_regs_ via antibody mediated cytotoxicity, as demonstrated in murine cancer models [40,41]. CTLA-4 inhibition is also able to broaden and remodel the peripheral TCR repertoire, as it was observed in cancer patients undergoing ipilimumab treatment [42]. Loss of CTLA-4 may lower the threshold for TCR ligation required for effective T cell activation since CTLA-4 normally acts to dampen TCR signal strength [25]. Blockade with either a-PD-1 or a-PD-L1 antibodies abrogates inhibition of TCR signaling and removes the brakes from T cells, unleashing their effector properties, while it also appears to be able to restore the function of exhausted T cells [43,44]. PD-1 blockade seems to be more effective in tumors infiltrated by tumor antigen-specific T cells that express PD-1 receptor but were kept in an inactive state due to the interaction of PD-1 with its ligands expressed by tumor cells and stromal cells within the TME [45].

## 3. ICI Therapy Failure and Tumor Immunogenicity

Although immunotherapy with ICIs holds much promise for durable outcomes in cancer regression and in some cases even cure, the majority of patients do not benefit by this course of treatment and either do not respond (innate/primary resistance) or relapse after an initial period of response (acquired/adaptive resistance) [46]. Emerging evidence from studies with patients treated with cancer immunotherapies indicates that the mechanisms of resistance broadly overlap with those normally used by cancers as they undergo immunoediting [47]. Several tumor cell-intrinsic and cell-extrinsic factors contribute to the resistance to therapy, leading to three different outcomes: i) insufficient generation of antitumor T cells, ii) inadequate function of tumor-specific T cells, and iii) impaired formation of T cell memory [46,48]. Amongst them, the immunogenicity of a tumor is considered to be a critical determinant of response to ICI therapy, as tumors devoid of tumor-specific antigens can never be recognized as foreign [49]. Anagnostou and colleagues examined the evolving landscape of tumor neoantigens during the acquisition of resistance to ICIs in non-small cell lung cancer (NSCLC) patients, and attributed this resistance to loss of mutations encoding cancer-specific neoantigens [50]. However, even a high mutational and neoantigen burden cannot lead to efficacious response if the patients’ cells lack a functional machinery for tumor antigen processing and presentation [46,51] since generation of reactive CD8+ T cells requires successful antigen processing and presentation of tumor-specific antigens [48].

## 4. Antigen Processing and Presentation in Cancer

For T cells to recognize malignant cells and attack them, two conditions are essential. First, the tumor cells have to report their intracellular changes on their surface and second, these changes must be sensed by T lymphocytes. The cellular mechanism that determines this direct interaction between the cancer and the adaptive immune system is the antigen processing and presentation pathway (APP) [52]. CD8+ T cells, via their TCR, can only detect aberrant cells in the context of peptide-MHC class I complexes. MHCI complexes are expressed on professional APCs that can activate naïve T cells, but also on all nucleated healthy and infected or transformed cells [53].

**Figure 1:**
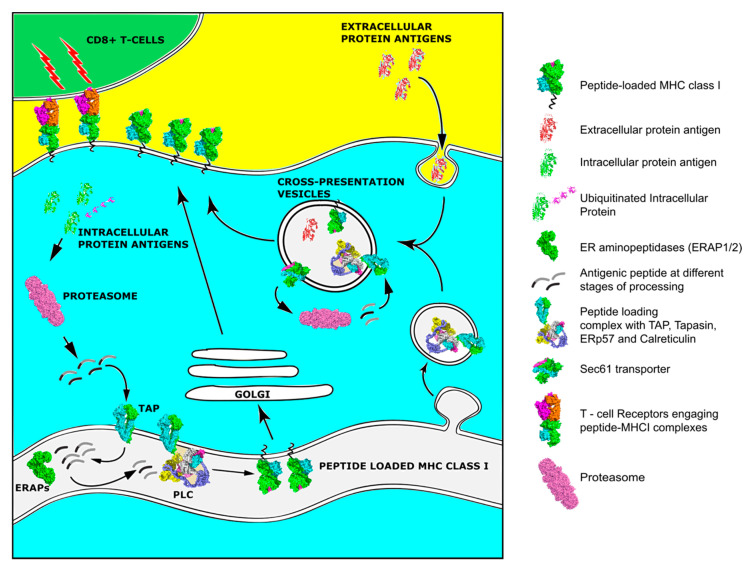
Overview of the Major Histocompatibility Class (MHC) class I pathway of antigen processing and presentation and the alternative pathway of cross-presentation.

For a peptide to serve as an epitope and therefore be capable of inducing an effective adaptive response, it has to be first processed by the cellular antigen processing machinery (APM) and then loaded onto an MHCI molecule (Figure 1). The processing and presentation pathway is a multi-step process in the context of the normal turnover of cellular proteins and often starts in the cytoplasm. There, intracellular proteins are ubiquitinated and fragmented into smaller pieces by the proteasome. An alternative pathway includes the proteasomal degradation of aberrant or misfolded proteins termed Defective Ribosomal Products (DRiPs) [54]. The constitutive proteasome is a barrel-shaped structure consisting of a catalytic 20S four-stacked ring core with chymotrypsin, trypsin, and caspase-like activities that is capped at each end by a regulatory 19S cap complex responsible for de-ubiquitination and unfolding of the trapped proteins that enter the main catalytic core. After exposure of the cells to inflammatory cytokines that generally enhance antigen presentation, new catalytic subunits named LMP2, LPM7, and LMP10 are produced and substitute these of the 20S proteasome to generate the immunoproteasome. This transition has been linked to changes in cleavage specificity, efficiency of MHCI ligand generation, and MHCI repertoire quantity [55]. Proteasomal cleavage generates peptides 2–26 residues long with a C-terminus anchor residue compatible with MHCI binding groove, but often extended at their N-terminus [55]. Peptides are then released in the cytosol and if they survive further degradation by cytosolic peptidases, are transferred into the endoplasmic reticulum (ER) by the Transporter associated with Antigen Processing, TAP. The TAP heterodimer, consisting from TAP1 and TAP2 subunits, forms a transmembrane pore in the ER membrane and preferentially transfers peptides 9–16 residues long, although longer peptides can be also transferred with much lower efficiency [56]. After a peptide enters the ER, its final assembly onto a nascent MHCI molecule is mainly orchestrated by a multi-subunit complex, called the Peptide Loading Complex (PLC) [57]. TAP constitutes an integral part of PLC, where it acts as a docking site for the MHC class I dedicated chaperone tapasin, and three other ER chaperones, the lectin calreticulin, calnexin, and the disulfide isomerase ERp57. Calnexin is important for early folding and oxidation of newly synthesized MHC heavy chain [56]. MHCI molecules are heterodimeric glycoproteins consisting of a polymorphic heavy chain (in humans encoded by the Human Leukocyte Antigen-HLA A, B, and C genes) and an invariable light chain, β2 microglobulin (β2m). MHCI molecules have a groove that can preferentially bind 8-11mer peptides. The exposed surface of this groove where the antigenic epitope is bound is the part of the MHCI complex that is recognized by the TCR [58]. Peptides that enter the ER and are too long to fit into MHCI are trimmed by the concerted action of two ER-resident aminopeptidases, ERAP1 and ERAP2 [59]. Calreticulin in combination with ERp57 assist with the folding and stabilization of the newly synthesized empty MHCI molecules. Tapasin mediates the recruitment of MHCI to the PLC and enables peptide loading and exchange, facilitating the formation of MHCI molecules with high affinity peptides. However, even after a peptide is loaded onto an MHCI molecule, an additional chaperone, TAP binding protein related protein, TAPBPR, assists with quality control to ensure peptide stable binding [60]. Having acquired a suitable peptide, the MHC class I molecule traffics to the cell surface through the Golgi network for presentation to T cells [56].

Tumors are particularly immunogenic and presentation of their specific antigens to T cells in an MHCI-restricted manner would lead to their eradication. Defects and alterations in the components of the APM are often found in tumors as cancer progression requires tumor cells to acquire the ability to avoid immune recognition [61]. Alterations in tumor APM can result not only in the downregulation of cell-surface expression MHCI molecules but can also alter the repertoire of antigenic peptides presented to the T lymphocytes. Since successful treatment with ICIs relies on re-activation of T cells, alterations in antigen processing and presentation of antigens can result to impaired antitumor responses and therapy resistance [16,62,63].

**Figure 2:**
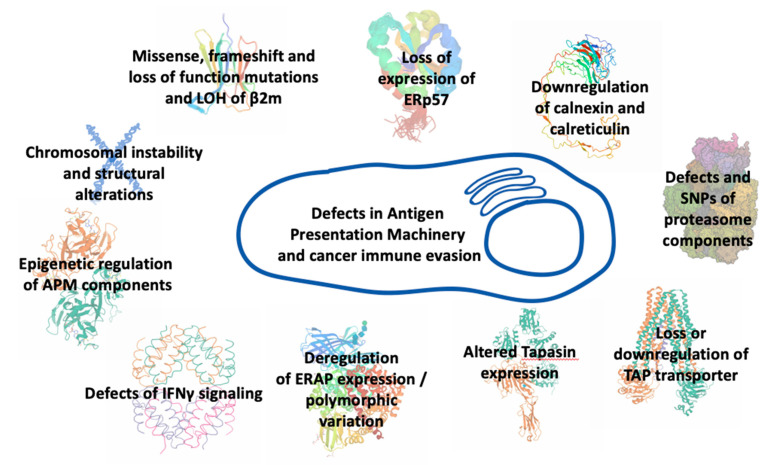
Defects in different components of the antigen processing and presentation machinery that can underlie immune evasion by cancer.

Alterations in antigen processing and presentation pathway may occur at any step of synthesis, assembly, transport, and surface expression of MHCI molecules or at any step of antigen editing (Figure 2). Truncating alterations, loss of heterozygosity, frameshift, and loss-of-function mutations affecting the β2m protein in human tumor cells that lead to instability of MHCI complexes, impaired folding, and diminished transport to the cell surface, have been associated with resistance to ICI therapy. In lung cancer patients, disruption of MHCI-mediated antigen presentation due to β2m loss of heterozygosity conferred resistance to PD-1 blockade therapy [64]. In melanoma metastatic patients treated with checkpoint inhibitors, point mutations, deletions, truncations, and loss of heterozygosity in β2m have been associated with resistance to ICI immunotherapy [16,62]. Furthermore, loss of expression of thiol reductase ERp57 has been demonstrated in several tumor types to correlate with poor prognosis [65,66,67]. Downregulation of calreticulin expression has been observed in colorectal and bladder cancers as well as in myeloproliferative neoplasms and has been associated with impaired antigen processing and presentation [68,69,70]. Defects have also been found in the IFNγ-inducible proteasome components [66,71,72]. Loss or downregulation of the transporter TAP have also been recorded in many cancer cell lines and primary tumors [72,73,74]. In all these cases, patients had a poor disease prognosis and diminished MHCI surface expression on tumor cells that correlated with changes in their antigenic peptide repertoire. In melanoma cells, micro-RNA downregulation of TAP expression led to reduction of MHCI surface expression and decreased T cell recognition [75]. In addition, the expression of Tapasin, another important APM component, has been found altered in several types of cancer [76,77,78]. MHCI surface expression was significantly decreased in all these patients and correlated with tumor progression. Impaired tapasin function led to MHCI molecules loaded with low-affinity, suboptimal antigenic epitopes. Furthermore, it reduced antigen presentation of tumor-specific antigens and blocked the presentation of certain immunodominant epitopes [77,78,79]. Mutations in tapasin and structural defects in IFNγ-related genes were found in recurrent metastatic melanoma with disease progression after active immunotherapy [80]. Furthermore, endoplasmic reticulum aminopeptidases, ERAP1 and ERAP2, exhibit variable expression levels in different cancer types [81]. Although mutations in these enzymes are rare, their expression in cancer is often either downregulated or upregulated while SNPs affecting their enzymatic activity can influence the immunopeptidome presented by MHCI molecules [82,83,84,85,86,87]. As these enzymes can both trim and destroy epitopes destined for binding onto MHCI molecules, their expression levels and activity strongly influence the peptide pool available for loading onto MHCI and can thus affect the immunogenicity of tumors [88,89]. In some cancers, ERAP1 overexpression leads to destruction of tumor-specific immunodominant epitopes and induction of anti-tumor CD8+ responses, linking antigen destruction with tumor escape [90,91,92]. In other cases, ERAP1 downregulation can lead to cancer rejection through Natural Killer cell mediated cytotoxicity [93,94]. ERAP2 overexpression in patients with oral cavity squamous cell carcinoma has been associated with metastasis from the primary tumor and poor prognosis [95], while the absence of ERAP2 in choriocarcinoma cells reduced their ability to activate T lymphocytes [96]. Downregulation of the mouse homologue, ERAAP, in mouse tumors increased the efficacy of a-PD1 blockade therapy [97]. In bladder cancer patients receiving a-PD1 therapy, expression quantitative trait loci affecting the expression of both ERAP1 and ERAP2 were found to associate with favored response to therapy and prolonged survival, probably due to alterations in the repertoire of peptides available for presentation to T cells [98]. Recently, functional ERAP1 allotypes have been correlated with tumor-infiltration by CD8+ T cells in cervical and oropharyngeal squamous cell carcinomas due to changes in processing of particular antigenic epitopes [99]. Overall, intracellular antigen processing is emerging as a master regulator of the immunogenicity of cancer [100,101].

As most components of the APP machinery are IFNγ inducible, defects in IFNγ signaling cascade can limit MHCI surface expression. The main proteins that interfere with this pathway are the transcription factors IFN-regulatory factor 1 (IRF-1) and STAT-1, and the kinases Janus-associated kinase JAK-1 and JAK-2. Tumor cells with activated IFNγ pathways can respond to cytokine secretion by immune cells located into the TME and become visible to T cells. Multiple studies have linked defects in IFNγ signaling with resistance to ICI therapy [16,17,102,103]. Genetic analysis of tumors from patients with melanoma and colon cancer who did not respond to PD1 blockade therapy despite their high mutation burden and high percentage of pre-existing tumor specific T cells, were identified to acquire loss of function mutations in JAK1/2 kinases and decreased MHCI surface expression [102,104]. Although indirectly associated to the IFNγ pathway, loss of the protein tyrosine phosphatase Ptpn2 was correlated to enhanced levels of antigen-loaded MHCI molecules on the surface of tumors and to sensitivity of tumor cells to immunotherapy [97].

Epigenetic events in cancer cells can regulate the expression of immune-related genes, resulting in changes in antigen processing and presentation that impair tumor recognition [105]. DNA methylation and histone modifications of MHCI heavy chain gene promoters leads to transcriptional silencing and decreased MHCI surface expression, causing impaired antigen presentation and immune evasion [106,107]. Additional components of the APM machinery have been found to be epigenetically regulated in many cancer types [107,108]. In melanoma, increased histone methyltransferase Ezh2 expression during a-CTLA-4 immunotherapy, decreased the antigen presentation ability of cancer cells while its inactivation reversed the resistance to therapy and synergistically suppressed tumor growth [109]. In prostate cancer, epigenetic silencing of the crucial component JAK1 kinase of the IFNγ signaling pathway led to IFNγ-insensitivity-mediated tumor evasion and resistance to immunotherapy [110]. Moreover, methylation of the NLRC5 MHCI trans-activator caused suppression of MHCI molecules and other components of the APM machinery in mice and an impaired ability to induce CD8+ T cell activation in cells [111]. In addition, Merkel cell carcinoma patients with low expression of APM components that was mediated by histone deacetylation, were resistant to a-PD1 therapy [112,113]. Antigen presentation efficiency is also diminished in human tumors characterized by large chromosomal instability and structural alterations. Although these tumors initially show induction of MHCI-restricted antigen presentation due to activation of cGAS/STING cytosolic DNA sensing pathway that detects tumor derived DNA and other pro-inflammatory signaling pathways, as they evolve under immune pressure, they suppress their antigen presentation machinery and adopt an immunologically poor phenotype. An experimental model of such tumor aneuploidy revealed that the suppression of antigen processing and presentation genes can be at least partly attributed to DNA hypermethylation of the corresponding genes, while the expression level of DNA methylotransferases was found significantly elevated [114].

## 5. MHCI Expression in Cancers

Downregulation of MHCI favors escape of tumor cells from immune surveillance [115]. Many studies in tumor cell lines and biopsies from patients reported total or partial loss of MHCI surface expression as a frequent event in cancer [72,116,117,118]. According to Garrido and colleagues, the loss or downregulation of surface MHCI expression is an active process that takes place gradually as tumors develop [119]. As such, at the early phase of tumor development, cancer cells are mostly MHCI positive. This induces T cell infiltration at the tumor microenvironment that recognize and kill cancer cells capable of presenting tumor-specific antigens on their MHCI molecules. Gradually, a vast diversity of tumor clones with variable MHCI surface expression levels is generated. A Darwinian type T cell-mediated selective pressure leads to tumors characterized by the presence of only MHCI negative cancer cells. This phase is accompanied by dramatic changes of the tumor tissue architecture that prevents T cells from entering the cancer niche as they are retained in the surrounding stroma [119,120]. This immune selection of MHCI-negative tumor cells has been demonstrated after immunotherapy in cancer patients and in experimental cancer models as the therapeutic application of checkpoint blockade increases the selective pressure towards tumor cells [121].

Apart from mutations, epigenetic modifications and structural alterations, tumors can adopt additional mechanisms to decrease their MHCI surface expression. A phenomenon often observed in tumors is the surface expression of non-classical MHCI molecules, such as HLA-G, HLA-E, and HLA-F. Although these molecules can present antigenic peptides in the context of antiviral defense, it is not clear whether their role in cancer is related to antigen presentation or they function as inhibitory ligands through their interaction with receptors on effector cells [122,123]. MHCI molecules can also be downregulated by other regulatory mechanisms, that involve signal transduction cascades, oncogenes, and tumor suppressor genes. Mutations in the BRAF oncogene (such as the V600E) lead to internalization of MHCI molecules from the cell surface of melanoma tumor cells and its sequestration within endocytic compartments, resulting in impaired recognition by the adaptive immune system [124]. In addition, autophagy, a conserved nutrient sensing system that induces the degradation of cytoplasmic proteins and damaged organelles by lysosomes can interfere with MHCI surface expression. In pancreatic ductal adenocarcinoma, Yamamoto and colleagues showed that MHCI molecules are selectively targeted for lysosomal degradation by an autophagy-dependent manner leading to alterations of immunogenicity of the tumor and impaired antigen presentation while its inhibition acts in synergism with ICI therapy and results in enhanced antitumor responses [125]. The SND1 oncoprotein, highly expressed in various cancers, prevents normal assembly of MHCI molecules by leading nascent synthesized MHCI heavy chain to ER-associated degradation (ERAD). Deletion of SND1 in tumor mouse models restores tumor antigen presentation to T cells both in vitro and in vivo and enhances T cell infiltration into the tumors [126]. Moreover, additional tumor suppressor genes (such as Fhit and p53) and oncogenes (such as Her2), interfere with MHCI expression in cancer cells [112,127,128]. In many human cancers, MHCI downregulation also associates with impaired signaling by transcription factors, such as NFkB and IRF2 that regulate activation of transcription of the MHCI heavy chain [112]. Additionally, IRF2 loss is associated with impaired peptide transport from the cytosol to the ER and peptide trimming [129]. Recently, it was demonstrated that the RNA binding protein MEX3B is linked with resistance to cancer immunotherapy in melanoma patients, by binding and destabilizing the HLA-A mRNA resulting in decreased HLA-A expression on the surface of tumor cells and thereby protecting the tumor cells by T cell-mediated recognition and elimination [130]. Finally, several long non-coding RNAs and miRNAs have been shown to modulate MHCI expression levels in several cancers [131,132,133,134].

In a recently published study, Chowell and colleagues analyzed the impact of individual’s specific MHC class I germline alleles on the clinical outcome of ICI therapy. By carrying out high-resolution MHCI genotyping of two patient cohorts with advanced melanoma and NSCLC that had received treatment with IC molecules, the authors observed that homozygosity in at least one human MHCI locus was linked to reduced survival periods, independently of mutational load, age, tumor stage, or type of therapy. Antigen presenting MHCI molecules are highly polymorphic, especially at their peptide binding grooves, and therefore each allele can bind and present a restricted set of antigenic epitopes. As result/Consequently, individuals homozygous in at least one MHCI locus may present a smaller, less diverse repertoire of tumor antigens to CD8+ T cells and thus may be less likely to present potent epitopes that induce highly effective antitumor responses that can be enhanced by ICI therapy [135,136]. Given that only a small percentage of presented tumor antigens in cancer patients are immunogenic, it seems that even small differences in MHCI molecules can significantly affect the adaptive responses and the efficacy of immunotherapy. This may also explain why MHCI homozygous patients with tumors bearing low neoantigen load show decreased survival and fail to respond to ICI therapeutic strategies compared to heterozygous patients and why specific HLA supertypes are associated with increased immune responsiveness. Moreover, Chowell and colleagues provided an additional link between MHCI heterozygosity and the presentation of a greater variety of tumor-specific antigens. By deep-sequencing of TCRs from tumor samples collected on-therapy, the authors observed enhanced clonality in heterozygous patients, concluding that the diversity of MHCI molecules modulates the selection and the resulting clonal expansion of T cells reactive against neoantigens and tumor-specific antigens after treatment with ICIs [137]. Accordingly, Marty and coworkers demonstrated that an individual’s MHCI genotype can predict cancer susceptibility as oncogenic mutations found in a tumor were linked to this genotype. Their study suggests that MHCI genotypes can act as a barrier that constrains the possible mutations that a developing tumor can accumulate [138].

## 6. Dendritic Cells and Cross-Presentation in Cancer

Although tumor cells often express MHCI molecules on their cell surface they tend to be poor antigen presenters and immune stimulators since they often lack costimulatory molecules and thus cannot effectively stimulate naïve T lymphocytes [139]. De novo generation and initiation of adaptive immune responses specific to tumor antigens, requires the cross-presentation capability of professional APCs that capture exogenous derived antigens, process and present them in order to prime naïve T cells [140]. The most potent known APCs are the dendritic cells (DCs), that constitute a heterogeneous cell population subdivided to several different subtypes [141]. DCs differentiate from bone marrow progenitors and reside in lymphoid and peripheral tissues where they act as sentinels of the immune system. Under steady-state conditions, differentiated DCs are found in their immature form. Immature DCs show a high endocytic potential and capture antigens but express low levels of MHCI and costimulatory molecules and as a result they do not prime T cells, but rather induce immune tolerance [142]. In order to be able to prime naïve T cells, DCs must first be activated and shift to their mature form. Their maturation is characterized by movement of MHCI to the cell surface, upregulation of the costimulatory molecules CD80 and CD86, higher expression of the C-C chemokine receptor 7 (CCR7), enhanced migration to lymph nodes (LNs), and increased cytokine production that drive T cell stimulation and clonal expansion [143,144,145]. DC activation is normally considered to result from detection of pathogen or damage associated molecular patterns (PAMPs/DAMPs) recognized by specific receptors. Within tumors, several of these receptors recognize endogenous DAMPs released or expressed on the surface of dead or dying cells. Immunogenic death of cancer cells, either spontaneously or due to therapeutic interventions, is an active process that releases alarmins and chemotactic factors that attract and activate DCs [146].

The inflammatory environment of a tumor, which includes cytokines, chemokines, and growth factors, fosters infiltration by DCs [147]. In tumors, DCs have access to large amounts of tumor antigens. After capturing and processing them through either the cytosolic or the vacuolar pathway, DCs migrate to the draining LNs to present these antigens and prime tumor-specific T cells. Memory and effector T cells return to the tumor site to perform surveillance and killing activities [148]. Studies with DCs isolated from tumor-bearing mice confirm their ability to cross-present tumor antigens and induce adaptive immune responses [149,150]. Apart from migratory DCs, non-migratory DCs that remain in the tumor may interact with T cells and prime them [147]. Additionally, by secreting IL-12 and other cytokines, non-migratory DCs can maintain and regulate antitumor responses. Antigen experienced T cells require cognate interactions with tissue DCs presenting antigens at a sufficient dose and duration to expand in situ and achieve their full effector activity [151]. Additionally, DCs in tumors can also be involved in priming of T cells, when found in ectopic or tertiary lymphoid structures in the immediate proximity of the tumor mass [152,153]. This phenomenon is especially important for the response against neoantigens that develop as tumor progresses [145]. Infiltration of DCs into tumor sites is associated with prolonged survival and reduced incidence of metastasis in patients with various types of solid tumors [154].

Cancer often develops evasion mechanisms that interfere with proper DC function [155,156,157]. In cancer patients, defective DC function is highly associated with impaired immune responses against antigens expressed by tumors [158]. The inherent plasticity of DCs and the balance between stimulatory and suppressive signals within the TME dictate whether DCs can induce and maintain a T cell response or not. In many cases, their number, distribution, phenotype, and function can change as the tumor progresses. Studies have shown that the number of DCs in peripheral blood of patients with head and neck squamous carcinoma is different from that of healthy individuals [159]. In a model of spontaneous ovarian cancer, Scarlett and colleagues observed a functional switch in DCs from an immunostimulatory to an immunosuppressive phenotype. Moreover, the depletion of DCs at early stages correlates to tumor growth while the depletion in later stages results in tumor regression. Finally, tumor DCs progressively upregulate PD-1 and PD-L1 and this phenomenon has been associated with T cell suppression and loss of Tumor infiltrating Lymphocytes (TILs) [160]. In the TME, DCs have either inefficient or totally absent antigen presenting capability or are polarized into immunosuppressive/tolerogenic regulatory DCs that suppress T cell activity [147,161,162]. The TME constitutes a challenging environment with limited availability of/for? oxygen due to poor vascularization and nutrients as well as increased concentration of metabolic products, which interfere with DC function, attenuating DC efficiency for cross priming [163]. A prerequisite for DC activation is their metabolic reprogramming to meet increased demands for protein synthesis and secretion of chemokines and cytokines that is accompanied by an increase in glucose uptake and enhanced levels of glycolysis. Competition for glucose uptake with other cells in the TME can render DCs unable to function properly [147]. The unique nature of the TME has also been highlighted by two separate recent studies that associated the impaired antigen presentation capacity of DCs with defects in trafficking of MHCI to the cell surface due to incorporation of tumor-derived oxidized lipids into DC lipid bodies. In this case, MHCI rather accumulate inside late endosomes [157,164]. Activation of the β-catenin pathway is another mechanism that cancer utilizes in order to inhibit cross-priming as activation of this pathway induces a tolerogenic state in DCs. Wnt ligands and other molecules, both in tumor cells and inside DCs, mediate DC exclusion from TME and inhibition of their antitumor activity, respectively. The DC intrinsic signaling route is also active in tumor infiltrating DCs in order to disrupt cross-presentation and reprogram DCs to induce tolerance [161]. In addition, many other factors (VEGF, IL-10, IL-6, colony stimulating factor CSF-1) inhibit maturation of bone marrow progenitors or monocytes into DCs, and instead drive monocytes toward a suppressive phenotype as they promote development of MDSCs and TAMs [165].

The clinical success of immunotherapy with checkpoint inhibitors relies significantly on effective processing and cross-presentation of tumor-specific antigens captured by DCs [158]. The blockade of inhibitory receptors on the cell surface of T cells by monoclonal antibodies, can intensify antitumor responses initially primed by DCs [166]. Tumor-bearing mice with impaired cross-presentation pathways showed resistance to therapy with antibodies targeting ICs [167,168]. During the last few years, new strategies have been emerging that aim to strengthen the therapeutic efficacy of checkpoint blockade treatment with DC-based vaccination, i.e., DCs loaded with tumor (neo)antigens for presentation to the immune system, as available preclinical and clinical data have demonstrated that DC-vaccination synergizes with ICIs for improved therapeutic outcomes [169,170]. In intracranial glioma tumor-bearing mice, the combined administration of an a-PD1 monoclonal antibody with a DC vaccine, led to long-term survival that was dependent on CD8+ T cells that infiltrated the tumor [170], while in a murine lung cancer model the combination of DC vaccination with ICIs led to 80% tumor eradication. The treated mice developed immunological memory that fostered cancer recurrence-free survival. In the same mice, monotherapy using either agent did not result in eradication of the tumor [171,172]. In human patients with active myeloma, the synergistic effect of the two therapies led to enhanced T cell responses against myeloma targets [173]. Ge and colleagues demonstrated that blocking the PD1/PD-L1 pathway with monoclonal antibodies, induces DC maturation and proliferation and that suppressing IC molecules during DC vaccination prolonged survival in a breast tumor-bearing mouse model [174]. Okada and colleagues used different MHCI-restricted tumor-associated neoantigens simultaneously with mature DCs and proposed that using this type of therapy at early stages of cancer can lead to generation of clinically useful neoantigen-specific T cells [175]. According to Linette and colleagues, vaccination using DCs appears to be necessary as an adjuvant to ICI therapy since most T cell clones specific for tumor neoantigens have been demonstrated to be naïve and below the limit of detection in patients with melanoma. In other words, a combinational therapy enhances both direct and cross-presentation and has the potential to boost the frequency and diversity of tumor-specific T cells and thus strengthen immune responses [176]. Finally, numerous studies have explored whether modulation of intratumoral APCs could increase the response to ICI therapies. These studies demonstrated that intratumoral DCs that sustain the potential to re-stimulate immune cells in the context of tumor microenvironment, are required for efficacious therapy outcomes, while their paucity limits the efficacy of ICIs [177,178,179].

## 7. The Immunopeptidome and Cancer

The sum of peptides bound and presented by MHCI on the surface of cells is increasingly referred to as the cellular immunopeptidome [180]. Under malignant conditions, the iummunopeptidome has been found altered both quantitatively and qualitatively. These altered tumor antigenic peptides may be recognized by the adaptive immune system as foreign and therefore induce immune responses. Indeed, in 2014, Gubin and colleagues first demonstrated that cancer immunotherapy treatments that boost T cell activity, overcoming tumor suppression induced by the tumor themselves, depend on T cell recognition of tumor-specific antigens [49]. Therefore, a detailed knowledge of the immunopeptidome constitution and deeper understanding of the characteristics of suitable tumor-associated rejection antigens can improve the current therapeutic immunotherapy interventions and offer new opportunities towards the development of personalized treatments. Several research efforts have already aimed at the identification of naturally presented antigens in different types of hematological and solid tumors while new tools and techniques (i.e., mass spectrometry-based and in silico, proteogenomic techniques etc.) are being developed and integrated, aiming for a more precise characterization and validation of the cancer-specific immunopeptidomes [181,182,183,184,185,186,187,188].

## 8. Tumor Antigens and Tumor-Associated Antigenic Peptides

A significant challenge in the field of immunotherapy is the identification of MHCI-presented peptides that are able to mediate T cell-based tumor rejection. Long-term clinical benefits of cancer immunotherapy treatments rely on T lymphocytes that recognize tumor antigens [189]. The major factors that determine whether an antigen is a good immunotherapy target are: (i) its immunogenicity, i.e., its ability to provoke an immune response after (re)-activation of T cells induced by ICIs, (ii) its tumor specificity, (iii) its prevalence and expression level on tumor cells, and (iv) its role in the oncogenic process [190].

Tumor antigens are classified into antigens of high tumoral and low tumoral specificity [189]. The first category includes antigens that are strictly tumor-specific, such as viral antigens generated in cancers of viral etiology and antigens derived from mutations or rearrangements in coding sequences and chromosome translocation events. The second group encompasses differentiation antigens, i.e., antigens expressed in both tumor and the corresponding healthy tissue but over-expressed in tumors [189]. A special category of tumor-associated antigens are RNA-editing derived epitopes. RNA editing is a posttranscriptional mechanism that generates sequence variations in proteins by enzymatic modification of nucleotides in mRNA sequences. This mechanism has been found dysregulated in cancers and peptides generated this way are presented to the immune system and elicit immune responses. In a recent study, Zhang and collaborators identified over-edited peptides from tumor tissues and provided evidence that effector CD8+ T cells specific for these peptides can be found in human tumors [191]. Moreover, potential tumor antigens can emerge after posttranslational modifications that occur on the antigens themselves and influence their binding affinity for MHCI [192,193]. Another class of antigenic peptides demonstrated to provoke immune responses and may constitute tumor rejection antigens, are the proteasome-generated spliced peptides [194]. In this case, distinct peptidic fragments (from the same protein or from different proteins) produced by the proteasome, are ligated *in situ*, producing sequences that are non-contiguous in the genome and are not found in proteins in the cell. In a recent study, Liepe and coworkers provided evidence that up to 30% of peptides bound to MHCI molecules can be spliced peptides [195]. However, this high prevalence of spliced peptides has been controversial and reanalysis of the original results by Mylonas and colleagues using multiple computational and verification tools estimated spliced peptides percentage to be much lower, in the range of 2–6% [196]. Finally, tumor antigens, named cryptic antigens, may also be derived from non-canonical translation of protein-coding genes or from translation of non-coding sequences. It has been proposed that up to 10% of the MHCI bound peptides can originate from non-coding genomic regions, untranslated regions and exonic out-of-frame translation. Additional sources of cryptic antigens can be long non-coding RNAs, altered mRNA splicing events, small nucleolar RNAs, and proteins encoded in ribosomal DNA [197]. If one takes into account that 99% of tumor-specific mutations are located in non-coding regions, these cryptic MHCI antigens can be a very rich source of tumor-specific antigens [197,198].

## 9. Epigenetic Control of Tumor Antigen Expression and Presentation

Tumor cells frequently exhibit epigenetic aberrations that significantly impact the repertoire of expressed proteins and therefore presented peptides, affecting recognition by immune cells. A class of tumor antigens that is epigenetically regulated and re-expressed in tumors is cancer testis antigens (CTAs). In healthy adults, CTAs are expressed only in male germ and trophoblastic cells [199]. However, ectopic expression has been observed in tumor cells of different histology—possibly indicating a role in oncogenesis and tumor growth—and is associated with global and promoter-specific DNA demethylation and histone modifications [115,199]. CTAs expressed by cancer cells are considered as tumor-specific antigens due to the fact that germ cells do not normally express MHCI molecules on their surface and additionally, due to the highly immunogenic capacity of CTAs. Indeed, potent cellular and humoral responses against these antigens, especially melanoma-associated MAGE and PRAME families and NY-ESO-1, have been observed in patients, while the use of demethylating agents in tumor cell lines increased their expression leading to recognition and destruction of the cancer cells by antigen-specific T cells [200,201].

Combination of ICI treatment with CTA vaccines has been demonstrated to have a synergistic positive effect. In melanoma patients, utilization of such a combinational treatment led to higher treatment response rates [202,203]. Immunological analysis showed that treatment with CTLA-4 immune-checkpoint antibody ipilimumab in metastatic melanoma patients enhanced NY-ESO-1 specific T cell responses and provided durable clinical benefits [204]. However, tumors can still find mechanisms to evade CTA-specific immune recognition and CTAs have been found downregulated in many cancers [202]. Moreover, dedifferentiated liposarcoma, leiomyosarcoma, and synovial sarcoma tumors with positive expression of PRAME cancer testis antigens have been demonstrated to reduce the expression levels of many components of the APP (such as MHC molecules, β2m, TAP2 and LMPs) in order to avoid immune recognition [205].

Apart from cancer testis antigens, other categories of antigens that could serve as tumor rejection antigens also appear to be under epigenetic regulation. Studies using DNA methyltransferase inhibitors demonstrated that these agents can induce the expression of transposable elements including mainly endogenous retroviruses (ERVs) [206]. ERVs are the most abundant viral elements in the human virome that are silenced due to DNA methylation in somatic cells [207]. Their activation in tumors results in a state of viral mimicry that can lead to generation of neoantigens in treated cancer cells. Their induced expression mimics exogenous retroviral infection and turns on viral defense genes resulting in innate immune responses, attraction of cytotoxic T cells in the TME, and IFNγ release that in turn induces transcription of APM components [207]. Indeed, a-PD-1 responsiveness has been positively correlated with ERVs expression in cancers [208]. Moreover, high molecular weight melanoma-associated antigens (HMW-MAAs) have been demonstrated to undergo demethylation at their gene promoter in melanoma lesions and cell lines, resulting in their re-expression, but whether these antigens can provoke immune responses remains elusive [209]. In a recent study, neoantigen expression levels were affected by promoter hypermethylation of genes harboring neoantigenic mutations in 23% of cases studied [11]. Qamra and colleagues analyzed chromatin profiles and the epigenomic promoter landscape in gastric adenocarcinoma and observed that epigenetically activated alternative tumor-specific promoters can favor immune evasion through depletion of immunogenic peptides and reduction of tumor antigenicity [210].

## 10. Neoantigens

Tumorigenesis and cancer outgrowth are closely related to genetic diversity and accumulation of non-synonymous somatic alterations. These alterations can be missense mutations, silent mutations, insertions, and deletions as well as copy number gains and losses that result in new peptide sequences which are strictly tumor-specific. A single alteration in amino acid sequence can interfere with T cell recognition in three different ways: (i) by creating an anchor residue that changes the binding affinity of the new peptide with the MHCI molecule; (ii) by changing the TCR binding properties resulting in a conformationally altered peptide-MHCI complex that can be recognized by different T cell populations and (iii) by altering processing of the protein by the cellular APM that could result to presentation of an epitope that normally would be degraded [211,212]. The number of mutations within a tumor genome is defined as tumor mutation burden (TMB). A high level of TMB raises the possibilities of generation of neoantigens and the emergence of neoantigens diverges cancer cells from normal, healthy cells. Cancer cells can now be recognized as foreign by the immune system as high levels of mutational load is believed to enhance antigen presentation to T cells and increase the chances of tumors being identified by widening the T cell killing repertoire [213,214]. The success of ICI therapy relies on reinvigoration of pre-existing T cells that although are kept under tight control by modulatory mechanisms, have the ability to recognize cancer cells and attack them when this control is unleashed [215]. Indeed, studies have demonstrated that neoantigens can elicit responses after immunotherapy treatment and T cells recognizing tumor-related neoepitopes have been identified in different cancers [216,217,218]. There is extensive published literature that correlates high mutation burden and neoantigen frequency with durable survival and regression benefit from/after ICI therapy in several types of tumors. Neoantigens have been proposed to be good predictive and prognostic markers of better clinical outcomes, although tumors with low mutational load can still respond to checkpoint blockade, indicating a non-linear correlation and the involvement of additional factors [219,220,221,222,223,224,225,226,227,228,229,230,231,232,233,234,235,236].

An important source of neoantigens comes from the accumulation of mutations that occur in the genome when the DNA repair mechanisms of the cell are deregulated. During the cell cycle, cells progress through a series of checkpoints before mitotic division to ensure replication fidelity. Cells are well equipped with mechanisms that recognize and correct DNA damages, such as proofreading polymerases, mismatch repair pathways, base and nucleotide excision pathways, and homologous repair mechanisms. However, tumors often develop defects in these mechanisms and, as a result, DNA replication errors accumulate leading to a large number of mutations that induce genomic instability, which in turn promote cancer growth. The major causes that drive repair deficiencies in cancer correlate with inherited and *de novo* germline and somatic alterations, at the DNA sequence level, in genes that constitute components of the repair machinery, as well as epigenetic alterations (DNA methylation, histone modifications, nucleosome remodeling, and RNA-mediated targeting) that lead to transcriptional silencing of the associated genes or changes in chromatin dynamics required for DNA repair [237].

Many studies have demonstrated the strong correlation between inactivation of DNA repair pathways and genomic instability with significant higher mutational burden, tumor neoantigen load, and immune cell infiltration [238,239,240,241,242]. Rospo and colleagues used a colorectal cancer model system and found that alterations in DNA repair genes facilitate the acquisition of dynamic neoantigen profiles that fluctuate over time [243] while similar results were also observed in lung squamous cell carcinoma [240]. A CRISPR/Cas9-mediated targeting of the mismatch repair (MMR) component *Mlh1* in murine breast, colon, and pancreatic ductal adenocarcinomas, revealed that MMR deficiency is associated with high mutational burden, TCR diversity, and significantly elevated neoantigen production. Furthermore, neoantigen production had continuous renewal potential compared to MMR-proficient cells that exhibited stable mutational load and neoantigen profiles [244]. The hyper-mutated phenotype that characterizes these types of tumors has been demonstrated to associate with higher rates of response to ICI therapy and durable clinical benefit [245,246,247,248]. In a study evaluating clinical data in patients with 12 different types of MMR-deficient tumors treated with an a-PD-1 agent, Le and colleagues observed rapid in vivo expansion of neoantigen-specific T cell clones reactive to mutant neoantigenic peptides found in the tumor. Such peptides may constitute a cohort of neoantigens useful for evaluating responses to IC treatment [247]. Collectively, it appears that there is growing evidence that the MMR deficient phenotype can serve as a good predictive biomarker of clinical response to ICI therapy.

## 11. T Cell Epitopes Associated with Impaired Peptide Processing

T cell epitopes associated with impaired peptide processing (TEIPP) constitute a unique, alternative repertoire of CD8+ T cell epitopes. TEIPP peptides are non-mutated self-antigens arising from housekeeping genes and emerge only in immune-edited tumors with low MHCI expression and defects in the APM as functional TAP seems to prevent their presentation. Their processing can also be conducted by alternative routes, such as the signal peptide route or the convertase family. TEIPP peptides are thought to be present within the ER of cells carrying intact TAP but cannot be presented due to their competition with the large flow of TAP-pumped peptides that are normally loaded onto MHCI molecules. A CD8+ T cell subset was discovered that selectively recognizes and targets tumor cells with defects in their APM and not cells with proficient APM. This T cell subset is positively selected in the thymus but remains in a naïve state in the periphery so it is not affected by tolerance [249,250,251]. The ppCT_16–25_ peptide derived from the signal peptide of pre-procalcitonin was the first human tumor epitope identified whose surface expression is associated with impaired TAP transporter function [252]. Moreover, in a recent study, 16 different HLA-A*02:01 presented TEIPP peptides were identified in mouse tumor models with defects in TAP transporter [253]. In addition, successful targeting of immune-escaped tumour variants by TEIPP-specific T cells was demonstrated [253]. TEIPP could be considered as tumor-specific neoantigens since their surface presentation is favored only under conditions of TAP dysfunction.

Recent work has highlighted that dysfunction of another APM component, ERAP1 (or ERAAP in mouse) can also lead to up-regulation of non-classical MHC class Ib molecules that normally present peptides from the signal sequence of MHCI [254]. Presentation by these non-classical MHC led to robust CD8+ responses [254]. Interestingly, ERAP1 downregulation affected the immunopeptidome of both classical and non-classical MHCI [255]. It was thus proposed that MHC class Ib presentation of signal sequence peptides may constitute a mechanism for immune surveillance for the dysfunction of the aminopeptidase trimming component of the APM [256].

## 12. Strategies for Enhancing ICI Therapy Effectiveness: The Role of Antigen Presentation

**Figure 3:**
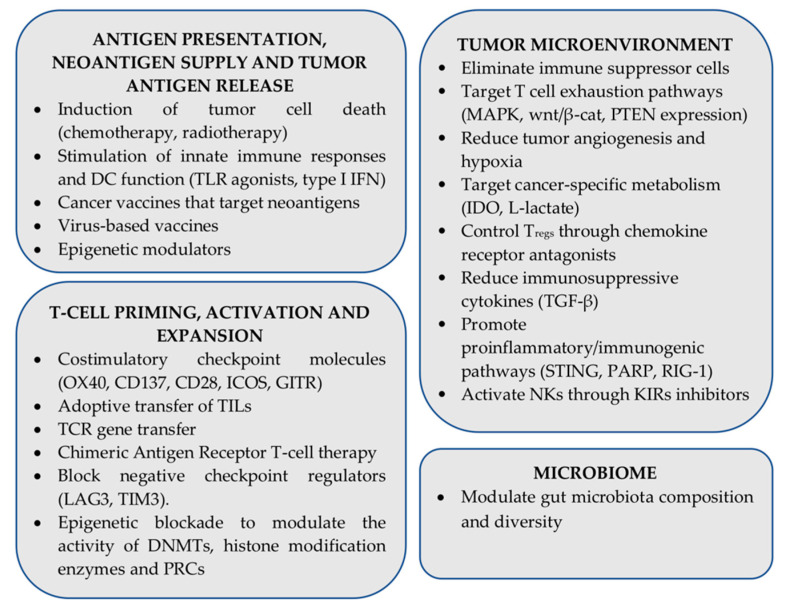
Combinatorial strategies under investigation that aim to enhance efficacy of immune checkpoint inhibitor (ICI) immunotherapy (PRC: Polycomb repressive complex).

Despite impressive clinical results, resistance to ICI immunotherapy is commonly a bottleneck in the successful treatment of several cancer types. Many approaches are currently under investigation aiming to surmount resistance to ICIs and improve clinical outcomes, often focusing on combining various therapeutic modalities (traditional therapies, other immunotherapy regiments as well as molecularly targeted therapies) on a checkpoint inhibitor backbone [257]. From a mechanism point of view, ongoing approaches aim to promote antigen processing and presentation, improve tumor antigen release and neoantigen supply, enhance T cell priming, expansion, survival, and effector functions, make the TME more friendly for immune cells, attenuate tumor-induced immunosuppressive factors, and promote proinflammatory/immunogenic pathways [258,259,260]. Moreover, there is significant evidence that gut microbiota diversity and composition can affect ICI responses and resistance in many cancers, by educating local and systemic immune responses, enhancing beneficial effects of metabolites, and dampening immune-related side-effects [261]. A summary of ongoing pre-clinical and clinical efforts focusing on overcoming resistance to ICI therapies is shown in Figure 3.

It is becoming increasingly clear that antigen processing and presentation is both central to cancer immune evasion and also a key puzzle piece in cancer immunotherapy. In order, however, to be able to manipulate APP to enhance cancer immunotherapy regiments, it is first necessary to understand the exact mechanisms by which APP is altered in cancer. Tumor cells can manipulate antigen presentation either by altering the cellular proteome or any of the components of the APP machinery. Therapeutically, several of the components of the APP machinery could be targeted in order to enhance the immunogenicity of cancer: the ubiquitin-proteasome degradation pathway, cytosolic peptidases, the TAP transporter, the peptide loading complex, peptide editing chaperones such as Tapasin or TAPBPR, ER aminopeptidases, and the MHCI themselves. In addition, induction of changes in the cellular proteome can regulate antigen presentation. In one study, researchers demonstrated a correlation between protein homeostasis and tumor antigen presentation by showing IFNγ-independent changes the MHCI peptide repertoire by low-level inhibition of the Heat Shock Protein Hsp90 [262]. Recently, Ilca and colleagues used a soluble form of the peptide editor TAPBPR and found an efficient way to bypass the peptides that are naturally presented and load onto tumour cells immunogenic peptides that resulted in robust immune responses [263].

One component of the APP that appears amenable to pharmacological targeting are the ER aminopeptidases ERAP1 and ERAP2 and the cross-presentation related aminopeptidase Insulin-regulated aminopeptidase IRAP. ERAP1 and ERAP2 appear to have a significant amount of specialization for antigen processing, whereas IRAP participates in additional biological processes including T cell receptor signaling [264]. Both ERAP1 and ERAP2 have been shown to be downregulated in some cancers [265], play key roles in the shaping of the cellular immunopeptidome [85], and their activity has been associated with changes in anti-cancer immune responses [101]. Furthermore, ERAP1 inhibitors have been shown to regulate the immunopeptidome [86] and elicit antitumor cytotoxic responses [90,91,94]. IRAP has been shown to be important in cross-presentation [266] and an IRAP inhibitor to be able to enhance cytotoxic responses ex vivo [267]. Furthermore, the development of inhibitors for these enzymes has reached significant maturity [268,269,270]. However, the synergism between inhibition of intracellular antigen processing by aminopeptidases and enhancement of antitumor immunity by ICI has not been explored yet.

**Figure 4:**
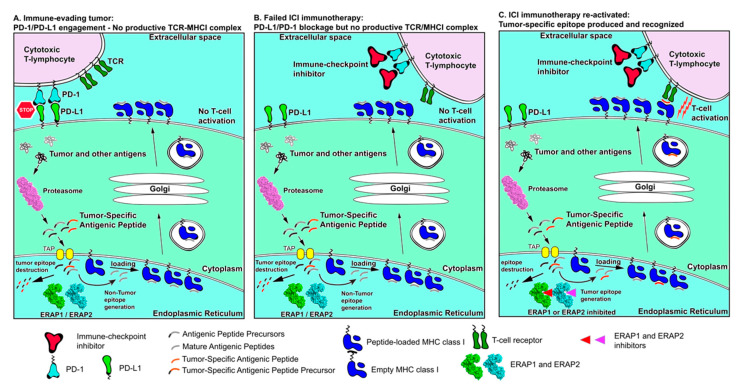
Schematic representation of antigen processing and presentation in cancer immune evasion and immune re-activation by ICIs and manipulation of intracellular antigen processing. (**Panel A**) tumor antigens are processed by the proteasome but then destroyed by ER aminopeptidases ERAP1 or ERAP2 resulting in lack of presentation on the cell surface. Overexpression of PD-L1 on the cancer cell surface downregulates cytotoxic T-lymphocyte responses. Synergism between these two mechanisms results in efficient immune evasion by the tumor. (**Panel B**) Immune-checkpoint inhibitors can help activate T cells but lack of tumor antigen presentation limits cytotoxic responses. (**Panel C**) inhibition of ERAP1 and ERAP2 can help rescue tumor-associated antigenic peptides from destruction and promote their presentation, which, in combination with ICI treatment, can help re-activate T cell cytotoxic responses against the tumor.

A potential synergism between aminopeptidase inhibition and ICI is depicted in Figure 4. As shown in panel A, an immune-evading tumor can be using ERAP1/ERAP2 to destroy tumor-associated antigenic peptides and over-expresses immune checkpoints such as PD-L1 to avoid T cell responses. Therapeutic interventions using ICIs, such as a-PD-1, can help promote T cell re-engagement but lack of presentation of appropriate tumor-associated antigenic peptides represents a bottleneck on antitumor cytotoxic responses (Panel B). Inhibition of ERAP1 or ERAP2, can help reactivate such responses by protecting tumor-associated antigenic peptides from degradation (Panel C). While this combinatorial approach is promising, it has not been experimentally evaluated and can suffer from a number of serious caveats since it cannot address exclusion of T cells from the TME or other means of T cell inactivation and antigen presentation silencing. Thus, it may be limited to specific cases or require combination with additional immunotherapy approaches. Given however the important role of APP in antitumor responses and the multitude of combinatorial cancer immunotherapy approaches currently under investigation, the modulation of intracellular antigen processing by aminopeptidase inhibitors is highly likely to find an application in enhancing tumor antigenicity.

## 13. Concluding Remarks

Expanding the benefits of cancer immunotherapy with ICIs to more patients and cancer types is probably one of the most urgent challenges in modern cancer therapy. The initial enthusiasm with ICI clinical successes gradually gave way to the realization that the interplay between the immune system and cancer is extremely complex and poorly understood. Many facets of this interplay have to synergize to circumvent the established evolutionary immune evasion of cancer. Not surprisingly, many immunotherapy approaches under investigation aim to combine multiple modulations of the immune system, including T cells and the TME, to achieve synergistic therapeutic effects. Antigen processing and presentation is undoubtably a key component in the immune evasion by cancer and thus its modulation constitutes a highly promising avenue for therapy. Still, being only one part of a larger puzzle, time will tell if manipulation of antigen presentation will be an effective monotherapy or it will find its place as a component of combination immunotherapy.

## Figures and Tables

**Table 1:** FDA-approved immune checkpoint inhibitors.

Name	Company	Target	Indications *
Ipilimumab (Yervoy^®^)	Bristol-Myers Squibb, New York, U.S.A.	CTLA-4	Melanoma, RCC, colorectal cancer, HCC, NSCLC, malignant pleural mesothelioma
Pembrolizumab (Keytruda^®^)	Merck Co., New Jersey, U.S.A.	PD-1	Melanoma, NSCLC, SCLC, HNSCC, urothelial carcinoma, primary mediastinal large B-cell lymphoma, gastric cancer, cervical cancer, esophageal cancer, TNBC, hepatocellular carcinoma, MCC, RCC, endometrial carcinoma, cutaneous squamous cell carcinoma, tumor mutational burden-High cancer, microsatellite instability or mismatch repair deficient colorectal cancer, microsatellite instability-High cancer
Nivolumab (Opdivo^®^)	Bristol-Myers Squibb, New York, U.S.A.	PD-1	Melanoma, RCC, NSCLC, SCLC, cHL, HNSCC, HCC, colorectal cancer, urothelial carcinoma, esophageal squamous cell carcinoma
Cemiplimab (Libtayo^®^)	Sanofi, Paris, France	PD-1	Cutaneous squamous cell carcinoma
Atezolizumab (Tecentriq^®^)	Roche, Basel, Switzerland	PD-L1	Urothelial carcinoma, NSCLC, TNBC, SCLC, HCC, melanoma
Avelumab (Bavencio^®^)	Pfizer, New York, U.S.A. and Merck, U.S.A.	PD-L1	Metastatic MCC, metastatic urothelial carcinoma
Durvalumab (Imfinzi^®^)	AstraZeneca, Cambridge, U.K.	PD-L1	Advanced or metastatic urothelial carcinoma, stage III NSCLC

* RCC: Renal cell carcinoma, HCC: Hepatocellular carcinoma, NSCLC: Non-small cell lung cancer, SCLC: Small cell lung cancer, HNSCC: Head and neck squamous cell cancer, cHL: classical Hodgkin Lymphoma, TNBC: Triple negative breast cancer, MCC: Merkel cell carcinoma.

## Data Availability

Not applicable.

## Abbreviations

APC	antigen presenting cell
APP(M)	antigen processing and presentation (machinery)
CTA	cancer testis antigen
CTLA-4	cytotoxic T-lymphocyte-associated protein 4
DC	dendritic cell
HLA	human leukocyte antigen
IC(I)	immune checkpoint (inhibitor)
LN	lymph node
MMR	mismatch repair
NK	natural killer cell
NSCLC	non-small cell lung cancer
PAMPs/DAMPs	pathogen/damage associated molecular patterns
PD-1	programmed cell death protein 1
MHCI	Major Histocompatibility Complex class I
TCR	T cell receptor
TIL	Tumor infiltrating Lymphocyte
TMB	tumor mutational burden
TME	tumor microenvironment
